# Less is more: relative rank is more informative than absolute abundance for compositional NGS data

**DOI:** 10.1093/bfgp/elae045

**Published:** 2024-11-20

**Authors:** Xubin Zheng, Nana Jin, Qiong Wu, Ning Zhang, Haonan Wu, Yuanhao Wang, Rui Luo, Tao Liu, Wanfu Ding, Qingshan Geng, Lixin Cheng

**Affiliations:** Guangdong Provincial Clinical Research Center for Geriatrics, Shenzhen Clinical Research Center for Geriatrics, Shenzhen People’s Hospital, Luohu District, Shenzhen 518020, China; Health Data Science Center, Shenzhen People's Hospital (First Affiliated Hospital of Southern University of Science and Technology), Luohu District, Shenzhen 518020, China; School of Computing and Information Technology, Great Bay University, Dongguan 523000, Guangdong, China; Guangdong Provincial Clinical Research Center for Geriatrics, Shenzhen Clinical Research Center for Geriatrics, Shenzhen People’s Hospital, Luohu District, Shenzhen 518020, China; Health Data Science Center, Shenzhen People's Hospital (First Affiliated Hospital of Southern University of Science and Technology), Luohu District, Shenzhen 518020, China; School of Basic Medicine, North Sichuan Medical College, Nanchong 637000, Sichuan, China; Guangdong Provincial Clinical Research Center for Geriatrics, Shenzhen Clinical Research Center for Geriatrics, Shenzhen People’s Hospital, Luohu District, Shenzhen 518020, China; Health Data Science Center, Shenzhen People's Hospital (First Affiliated Hospital of Southern University of Science and Technology), Luohu District, Shenzhen 518020, China; Guangdong Provincial Clinical Research Center for Geriatrics, Shenzhen Clinical Research Center for Geriatrics, Shenzhen People’s Hospital, Luohu District, Shenzhen 518020, China; Health Data Science Center, Shenzhen People's Hospital (First Affiliated Hospital of Southern University of Science and Technology), Luohu District, Shenzhen 518020, China; Guangdong Provincial Clinical Research Center for Geriatrics, Shenzhen Clinical Research Center for Geriatrics, Shenzhen People’s Hospital, Luohu District, Shenzhen 518020, China; Health Data Science Center, Shenzhen People's Hospital (First Affiliated Hospital of Southern University of Science and Technology), Luohu District, Shenzhen 518020, China; Department of Systems Engineering, City University of Hong Kong, Kowloon, Hong Kong SAR; International Digital Economy Academy (IDEA), Futian District, Shenzhen 518020, China; Guangdong Provincial Clinical Research Center for Geriatrics, Shenzhen Clinical Research Center for Geriatrics, Shenzhen People’s Hospital, Luohu District, Shenzhen 518020, China; Guangdong Provincial Clinical Research Center for Geriatrics, Shenzhen Clinical Research Center for Geriatrics, Shenzhen People’s Hospital, Luohu District, Shenzhen 518020, China; Guangdong Provincial Clinical Research Center for Geriatrics, Shenzhen Clinical Research Center for Geriatrics, Shenzhen People’s Hospital, Luohu District, Shenzhen 518020, China; Health Data Science Center, Shenzhen People's Hospital (First Affiliated Hospital of Southern University of Science and Technology), Luohu District, Shenzhen 518020, China

**Keywords:** pairwise analysis, relative expression, compositional data analysis, data integration, transcriptome

## Abstract

High-throughput gene expression data have been extensively generated and utilized in biological mechanism investigations, biomarker detection, disease diagnosis and prognosis. These applications encompass not only bulk transcriptome, but also single cell RNA-seq data. However, extracting reliable biological information from transcriptome data remains challenging due to the constrains of Compositional Data Analysis. Current data preprocessing methods, including dataset normalization and batch effect correction, are insufficient to address these issues and improve data quality for downstream analysis. Alternatively, qualification methods focusing on the relative order of gene expression (ROGER) are more informative than the quantification methods that rely on gene expression abundance. The Pairwise Analysis of Gene expression method is an enhancement of ROGER, designed for data integration in either sample space or feature space. In this review, we summarize the methods applied to transcriptome data analysis and discuss their potentials in predicting clinical outcomes.

## Introduction

The analysis of gene expression data from Next Generation Sequencing (NGS) technologies is fundamental to biological mechanism investigations, biomarker detection, disease diagnosis, and prognosis [1–6]. Compositional data analysis (CoDA) [7] has recently emerged as a new paradigm for the analysis of NGS data, including transcriptomics (RNA-seq and single-cell RNA-seq), metabolomics, proteomics, lipidomics, 16S rRNA gene sequencing, and metagenomic sequencing [8–10]. NGS data are compositional, in the sense that the library size of a sequenced sample is constrained to a constant without any biological meaning [7].

Typically, read counts are preprocessed using normalization algorithms to make the gene expression abundance comparable across samples. However, these algorithms often fail to account for the compositional nature of NGS data. Compositional data refers to a type of data where the numbers make up proportions of a whole, meaning the components not independent since their sum is arbitrarily limited [7, 11]. Compositional data exist in a simplex, a spatial configuration defined by dimensions corresponding to the components of the data. This type of data is prevalent, not limited in NGS data, but also in other domains, such as voting data, nutrient information on food labels, gas proportions in the air, and even the red, green, and blue values on the pixels of our monitors.

Two main strategies for preprocessing NGS compositional data: 1) normalization to recover absolute abundance of read counts; and 2) CoDA methods that transform the data using within-sample references, such as Centered Log-Transformation (CLR) and Additive Log-Transformation (ALR) [12]. Normalization is frequently employed when identifying differentially expressed genes (DEGs) [13–15]. However, its effectiveness may be compromised if RNA quantities vary significantly across samples [16]. CoDA uses log-ratio transformation to avoid assumptions of traditional normalization algorithms, but choosing a reliable in-sample reference remains challenging. ALR and CLR are commonly used in CoDA, transforming data from the simplex space to Euclidean space for standard analysis [7, 12].

Several algorithms have been developed for detecting expression changes in compositional data across different conditions, such as ALDEx2 [17] and ANCOM [18]. However, most transcriptome studies mostly rely on conventional algorithms like DESeq2 [19] and EdgeR [20], which do not account for the compositional nature of the data. DESeq2 and EdgeR assume that the absolute abundances are identical among different conditions, akin to the CLR transformation used in CoDA methods. CLR transformation uses the geometric mean of the genes as a reference, meaning that all results must be interpreted with respect to the geometric mean. The underlying assumption is that the geometric mean of the genes in each sample does not change between conditions. This assumption often leads to significant changes in the absolute counts, which is a frequent pitfall encountered by researchers [14, 15, 21].

Moreover, spike-in normalization, which introduces external references to address normalization issues, is effective but requires careful reference setup that can be difficult to achieve in practice [7]. The closure effects inherent in biological systems and the library preparation process can lead to inaccuracies if not properly accounted for. Similarly, CoDA methods, which employ relative value transformations to avoid the assumptions of traditional normalization algorithms, face challenges in selecting reliable within-sample references [22]. In addition, biological systems themselves could be inherently compositional, for instance, a cell may produce only a limited amount of RNAs, in such cases, no external references are introduced outside the cell to adjust the true absolute counts.

However, these traditional normalization algorithms in gene expression analysis often assume that the total RNA content is consistent across samples, which is not always the case in practice. These algorithms aim to correct technical biases such as differences in sequencing depth or RNA extraction efficiency by scaling or transforming the data to make it comparable across samples. However, this approach can introduce its own set of assumptions and potential biases, particularly when dealing with compositional data where the total RNA content is constrained by the library size and does not reflect biological variability.

In contrast, the relative rank methods focus on the relative order of gene expression within individual samples. These non-parametric approaches avoids the assumptions inherent in traditional normalization methods by not relying on the absolute abundance of transcripts. Instead, they leverage the stability of gene expression ranks, which are less susceptible to systematic biases and batch effects. By comparing the relative ranks of genes, relative rank-based methods can identify differential expression patterns without the need for extensive normalization procedures, thus providing a more robust and unbiased analysis of gene expression data.

**Figure 1:**
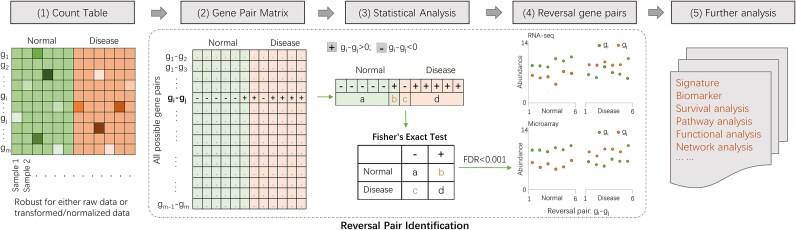
The core workflow of ROGER and PAGE. (1) An input count table with rows representing genes and columns representing samples. (2) A gene pair matrix including the expression relative rank of all possible gene pairs. + (−) represent the gi is larger (lower) than gj. (3) a and c represent the number of samples with the expression level of gi < gj in normal and disease samples, while b and d represent the number of samples with the expression level of gi > gj in normal and disease samples, respectively. The Fisher’s exact test is used to test the significance of the rank difference of gi and gj between conditions. The P values are adjusted by Benjamini-Hochberg multiple tests with a given FDR threshold is defined as differentially ranked. (4) Illustration of the identified differentially ranked gene pairs in RNA-seq and microarray data, respectively. (5) Further analysis based on the identified differentially ranked gene pairs.

Therefore, alternative methods such as **ROGER and PAGE** have been developed to focus on the relative order of gene expression and the relative expression between gene pairs, respectively (Fig. 1 and Table 1). These methods (see Table 1) are independent of traditional normalization assumptions and preprocessing techniques, thereby mitigating technical biases and providing robust, biologically meaningful signals for downstream analysis.

**Table 1:** Preprocessing methods for CoDA.

**Tool**	**Brief introduction**	**Reference**
RankComp	RankComp was developed to identify DEGs in individual samples by analyzing the highly stable REOs landscape observed in normal samples and comparing them with the disrupted REOs in the disease samples.	[11]
RankCompV2	The rank-driven signatures, in contrast to the abundance-driven signatures, are claimed to be applicable individually for independent clinical samples. While RankCompV2 exhibits higher statistical power compared to RankComp, it demonstrates lower accuracy.	[12]
LncRIndiv	LncRIndiv was developed to establish an individual-level prognostic lncRNA signature specifically for lung adenocarcinoma.	[14]
RankMiRNA	The RankMiRNA algorithm demonstrates high accuracy in identifying differentially expressed miRNAs at the individual level.	[15]
iPAGE	The Individualized Pair Analysis of Gene Expression (iPAGE) method was utilized to identify gene pairs as potential biomarkers.	[23–30]
GPGPS	The GPGPS is an ensemble gene pair signature that was developed for risk assessment and survival prediction in glioma.	[23]
MrGPS	The MrGPS, developed using the iPAGE algorithm, effectively integrates m6A-related gene expression patterns to provide a robust prognostic tool for glioma patients.	[24]
scPAGE	scPAGE, built upon the iPAGE framework, leverages the power of single-cell and bulk transcriptome data and successfully classifies sepsis patients from non-sepsis individuals.	[26]

### Identifying differential expression genes using ROGER

An alternative to spike-in normalization and CoDA transformation is the use of relative rank methods, which do not require external references and focus solely on the relative ranks rather than absolute abundance. Guo *et al.* developed a series of relative rank methods [31–39], concentrating on the **ROGER** within individual samples. It has been reported that the within-sample rank of genes is not affected by systematic biases and batch effects in microarray and RNA-seq data [39]. Consequently, ROGER methods are independent of preprocessing steps such as intensity normalization, compositional transformation, and batch effect correction. Here, we summarized an overview of **ROGER** and briefly introduce five methods based on this approach.

Mathematically, ROGER is grounded in the concept of relative ranks, which are used to compare the expression levels of gene pairs across samples. The method operates on the principle that the relative rank of genes is consistent across samples and is robust to systematic biases and batch effects.

Algorithmically, ROGER involves the following steps (as illustrated in Fig. 1):

1. Construction of a gene pair matrix from the input count table, where each entry represents the relative rank of a gene pair within a sample.

2. Application of statistical tests, such as the Fisher’s exact test, to determine the significance of the rank differences between gene pairs across conditions.

3. Adjustment of p-values to account for multiple testing, typically using the Benjamini-Hochberg procedure to control the false discovery rate (FDR).

4. Identification of differentially ranked gene pairs as those with a significant rank difference (FDR < 0.05).

The ROGER method has been implemented in various studies, demonstrating its effectiveness in identifying differentially expressed genes without the need for traditional normalization methods. Its independence from preprocessing steps such as intensity normalization, compositional transformation, and batch effect correction makes it a versatile and robust tool for transcriptomic data analysis.

***RankComp*** has recently emerged as a new paradigm for the differential analysis of transcriptome data [31]. unlike traditional approaches that rely on elaborate normalization procedures to make read counts or expression intensity comparable across samples, **RankComp** utilized **ROGER,** thereby avoiding the assumptions associated with normalization. In other words, these algorithms are applicable to all types of data, whether relative or absolute, compositional or not. RankComp assumes that the **ROGER** in control samples is relatively stable, whereas in disease tissues, it may change substantially to reveal patient-specific differential information. Genes in each sample are ranked according to their expression values, either increasingly or decreasingly. Pairwise comparisons are then performed for all genes to identify stable gene pairs, which are a pair of genes with stable order in normal samples, and reversal pairs, which are gene pairs with opposite order in disease samples compared to the stable pairs. **RankComp** identifies DEGs in individual samples by analyzing the highly stable relative expression orders (REOs) landscape observed in normal samples and comparing them with the disrupted REOs in disease samples.

***RankCompV2*** separately screens stable gene pairs using ROGER in breast cancer samples and corresponding normal samples [32]. The common stable gene pairs of the two groups with reversal direction are first identified, followed by Fisher’s exact test for differential analysis. The update in **RankCompV2** involves a gene pair filtering process that excludes gene pairs containing potential DEGs in both consistent and reversal gene pairs. In contrast, **RankComp** only excluded gene pairs with potential DEGs in the opposite direction in reversal gene pairs, which may decrease statistical power. **RankCompV2** has higher statistical power compared to **RankComp**, although it has lower accuracy. The statistical power in the context of RankComp and RankCompV2 is influenced by the filtering of gene pairs. RankComp's approach to excluding only gene pairs with potential DEGs in the opposite direction within reversal gene pairs may inadvertently retain gene pairs that contribute noise to the analysis. This can dilute the signal and decrease the ability to detect true DEGs, thus reducing statistical power. RankCompV2 addresses this by implementing a more stringent gene pair filtering process that excludes gene pairs containing potential DEGs in both consistent and reversal gene pairs. This more rigorous filtering helps to reduce the variance in the data and increases the signal-to-noise ratio, leading to higher statistical power. The improved power of RankCompV2 is due to its ability to focus the analysis on gene pairs that are more likely to be biologically relevant and less affected by random fluctuations in gene expression.

They also applied **ROGER** to extract a prognostic gene pair signature in a case of non-small-cell lung cancer [33]. Typically, prognostic gene signatures for diseases are based on risk scores, traditionally calculated as a polynomial of gene abundance from a set of genes. However, these signatures are not robust across different preprocessing procedures. Unlike abundance-driven signatures, rank-driven signatures claimed to be applicable to independent clinical samples individually.

***LncRIndiv*** was developed to identify a prognostic long non-coding RNA (lncRNA) signature at the individual level for lung adenocarcinoma [34]. It defines a stable pair as a pair of lncRNAs (A and B) with a consistent order (A > B or A < B) across more than 95% of normal samples. A reversal pair is defined as a pair of lncRNAs that show a significantly high proportion of opposite direction among cancer samples compared to the stable pair. Statistical significance is tested using Fisher’s exact test with FDR < 0.1. An elaborate approach was then designed. For every reversal pair partner of a specific lncRNA (say A), the coefficient of variation (CV) of rank across cancer and normal samples was calculated. It is hypothesized that if the ranks of partners are constant across samples (small CV), the reversal between A and its partner is mainly due to the rank change of A, indicating that A is differentially expressed in individual cancer samples. lncRNA A is defined as differentially expressed if more than half of the reversal pairs are detected in an individual.

Furthermore, leveraging ROGER, they proposed an algorithm called ***RankMiRNA*** to identify differentially expressed miRNAs for individual cancer tissues [35]. They successfully identified and validated three common DE miRNAs that are dysregulated in over 90% of lung cancer samples, along with several subtype-specific miRNAs for lung cancer. **RankMiRNA** effectively identifies DE miRNAs at the individual level.

### Unified Data Integration across platforms and datasets using PAGE

***iPAGE*** (Individualized Pairwise Analysis of Gene expression) is a novel algorithm for feature selection and data integration based on relative RNA expression [23–30]. iPAGE leverages the relative expression levels between each pair of RNAs within samples to retrieve differential RNA pairs, which are reversely expressed in disease, e.g. RNAi is greater than RNAj in normal controls but less than RNAj in disease patients. By using relative expression rather than absolute expression levels, iPAGE reduces the impact of technical variation and systematic experimental bias. This algorithm also enables cross-platform analysis since it relies on the relative relationships of gene pairs within samples rather than on platform-specific absolute expression levels. Furthermore, iPAGE has demonstrated robust performance across cohorts of different age groups and various normalization methods, proving its robustness.

The **SepSigLnc** signature, developed by Zheng *et al.* using the iPAGE algorithm, has demonstrated outstanding performance in distinguishing sepsis patients from normal controls [29]. By integrating whole blood lncRNA expression profiles from sepsis patients, the researchers identified 14 lncRNA pairs that form the SepSigLnc signature. This SepSigLnc signature has proven to be high accurate and reliable in sepsis diagnosis, consistently showing excellent performance across multiple independent cohorts. Notably, when compared to common machine learning models and existing signatures, the SepSigLnc signature outperformed them in the validation cohorts of the same age group, achieving AUC (area under the curve) values of 0.990 and 0.995. furthermore, it demonstrated an average AUC of 0.878 across different groups and an average AUC of 0.953 in cohorts processed with an alternative normalization method. These results suggest that the SepSigLnc signature, developed using the iPAGE algorithm, is a robust and reliable tool for diagnosing sepsis, surpassing existing approaches in terms of accuracy and reliability.

The **GPGPS** (Glioma Prognostic Gene Pair Signature) is an advanced ensemble gene pair signature designed for risk assessment and survival prediction in glioma [23]. this signature was constructed based on prior knowledge of IDH and 1p/19q status. Cheng *et al.* developed two distinct gene pair signatures, the IDH-GPS and the 1p/19q-GPS. These signatures serve as valuable transcriptome markers mirror the corresponding genomic variations in glioma. by integrating these two models, GPGPS demonstrated superior performance, showing higher AUC values for overall survival prediction in glioma compared to individual GPSs and other existing prognostic signatures. This study underscores the power of converting genetic alterations into expression changes to facilitate robust prognostic investigations in glioma. The GPGPS, as an ensemble of the IDH-GPS and 1p/19q-GPS, illustrates the significant potential of leveraging both genomic and transcriptomic information to improve risk assessment and survival prediction for glioma patients.

The **MrGPS** (m6A-related Gene Pair Signature) was developed by Zhang *et al.* using the iPAGE algorithm to assist in glioma prognosis [24]. Initially, the researchers paired 30 m6A regulators with their target genes, forming m6A-related gene pairs. m6A methylation, a prevalent RNA modification, is linked to various cancer developments. The researchers collected transcriptome data from 529 low-grade glioma (LGG) and 168 glioblastoma (GBM) patients from TCGA, identifying 122 m6A-related gene pairs with significantly reversed relative expression values between LGG and GBM. Using Least Absolute Shrinkage and Selection Operator (LASSO) Cox regression analysis, they constructed a prognostic model incorporating five gene pairs, termed MrGPS. This model was validated in independent CGGA and GEO glioma cohorts. Notably, MrGPS outperformed existing glioma prognostic models, demonstrating superior accuracy and robustness. The iPAGE algorithm’s integration of data across different platforms, overcoming batch effects and data normalization issues, contributed to its success. In summary, MrGPS, through the iPAGE algorithm, effectively combines m6A-related gene expression patterns, offering a robust prognostic tool for glioma patients.

The **scPAGE** (single-cell Pair-wise Analysis of Gene Expression) is an innovative computational method developed to differentiate sepsis patients from non-sepsis individuals [26]. Building on the iPAGE framework, which demonstrated the effectiveness of gene expression pairwise relationships in phenotype classification, scPAGE leverages both single-cell and bulk transcriptome data. Specifically, it focuses on single-cell RNA sequencing (scRNA-seq) data, identifying gene pair signatures that could effectively distinguish between cells from septic and non-septic patients. This single-cell-based approach is highly generalizable, performing well even in bulk RNA-seq datasets. The key innovation of scPAGE lies in its ability to harness the information encoded in gene expression pairwise relationship, rather than relying solely on individual gene expression levels. By identifying the gene pairs showed statistically significant reverse expression pattern between phenotypes, scPAGE developed a robust classification model for separating sepsis patients from non-sepsis individuals. The successful application of scPAGE to both single-cell and bulk RNA-seq data highlights its versatility and potential for broader use in diverse biomedical applications beyond sepsis classification. This demonstrates the power of integrating single-cell and bulk transcriptomic approaches to uncover novel, clinically relevant signatures.

### Consensus and controversy on ROGER and PAGE

The ROGER-based and PAGE-based methods represent a series of innovative approaches that focus on the relative order of gene expression within individual samples. These methods are designed to overcome the limitations of traditional normalization techniques by leveraging the stability of gene expression ranks across samples. For example, scPAGE performs superior to linear regression, multilayer perceptron, random forest, support vector machine, and ridge regression in various data sets [26]. The biological applications and clinical insights offered by ROGER and PAGE are manifold, encompassing the identification of patients across a spectrum of diseases, including various forms of cancer and sepsis. These tools are also instrumental in predicting prognosis, assessing risk, and stratifying patient populations. To provide a clearer understanding of the statistical underpinnings of these methods, we have included a brief explanation of the key statistical tests employed. The Fisher Exact Test is a statistical method used to determine the significance of associations between two categorical variables in a sample. It is used to determine the significance of the rank difference between gene pairs across different conditions. It is particularly useful for analyzing contingency tables with small sample sizes, which is often the case in gene expression studies. LASSO Cox Regression Analysis is employed for survival analysis and feature selection, which is to identify a set of significant predictors that are most related to patient prognosis. By imposing a penalty on the regression coefficients, LASSO helps to reduce the complexity of the model and prevent overfitting, which is crucial when dealing with high-dimensional gene expression data. These methodological details and statistical explanations aim to enhance the transparency and reproducibility of our research, enabling readers to fully grasp the analytical framework of the ROGER-based methods.

While the ROGER and PAGE methods offer significant advantages over traditional normalization techniques, it is important to acknowledge their potential limitations. Both methods are predicated on the stability of gene expression ranks across samples, which may not always hold true, particularly in the presence of extreme variability or small sample sizes. Additionally, the ROGER method assumes that the relative order of gene expression is more informative than absolute abundance, which may not be the case for all biological contexts.

Furthermore, both methods rely on the assumption that the relative rank or the relative expression between gene pairs is biologically meaningful and consistent across different conditions. If this assumption does not hold, the results could be misleading. It is also crucial to consider that the interpretation of results from ROGER and PAGE may require researchers to shift from their habitual mindset of absolute expression levels, which could pose a learning curve for some users.

Despite these limitations, the ROGER and PAGE methods represent valuable tools for gene expression analysis, offering a fresh perspective on transcriptomic data that can uncover insights not readily apparent with traditional methods. Future research should focus on addressing these limitations and on further validating the applicability of these methods across diverse biological datasets.

The computational complexity of the ROGER and PAGE methods is an important consideration when evaluating their practicality for large-scale studies. Traditional normalization methods often require extensive computational resources, especially when dealing with large datasets, due to the iterative nature of the algorithms and the need for multiple statistical tests.

In contrast, the ROGER method, by focusing on the relative order of gene expression within samples, significantly reduces the computational burden. Since it does not require extensive normalization or transformation steps, ROGER is computationally efficient, making it suitable for large-scale gene expression analysis.

The PAGE method, while more sophisticated in its approach to data integration, also maintains a reasonable computational complexity. By leveraging the relative expression between gene pairs, PAGE avoids the need for complex modeling of gene expression levels, which can be computationally intensive. This makes PAGE a practical choice for large-scale studies, where it can be used to integrate data from multiple sources without incurring prohibitive computational costs. Further research should be directed towards benchmarking and comparing the computational efficiency of ROGER and PAGE with that of traditional methods.

The PAGE method represents a significant advancement over the ROGER method, offering enhanced capabilities for data integration and analysis [40]. PAGE is designed to leverage the relative expression between gene pairs, contrasting with ROGER's approach of concentrating on the relative ranking of gene expressions, providing a more subtle view of gene expression dynamics that can be particularly powerful in the context of complex biological systems.

The specific innovations and advantages of the PAGE method over ROGER include:

1. Data Integration: PAGE is particularly adept at integrating gene expression data across different platforms and datasets. This capability is crucial for meta-analyses and for leveraging the wealth of transcriptomic data available from diverse sources. 2. Robustness to Data Heterogeneity: By focusing on the relative expression between gene pairs, PAGE is robust to the heterogeneity that often characterizes large-scale gene expression studies. In large-scale genomic studies, samples are often sourced from multiple laboratories and subjected to diverse processing protocols, which can result in varying scales of gene expression data. PAGE excels in such scenarios by focusing on the relative expression levels between genes rather than the absolute expression levels of individual genes across samples. 3. Biological Interpretability: A gene pair identified using PAGE are usually included in a same pathways or same functional terms, which can provide insights into the functional relationships between genes. For instance, rather than pairing genes arbitrarily, the authors conducted pair extraction within each specific biological pathway when constructed bvnGPS and bvnGPS2 [25], offering a more specific and biological interpretation of the qualified gene expression data.

In summary, both ROGER and PAGE offer efficient alternatives to traditional normalization methods, making them well-suited for large-scale gene expression studies. Their ability to reduce computational complexity without compromising the accuracy of the results positions them as valuable tools for high-throughput data analysis in genomics research.

## Conclusion

NGS RNA-seq data are compositional because the total number of reads sequenced for a sample is constrained to an arbitrary limit that lacks biological significance. Conventional normalization algorithms, designed to obtain absolute abundance, are theoretically unsuitable in this context. Moreover, CoDA methods that utilize log-ratio transformation are impractical due to its high dependence on external references.

The ROGER-based and PAGE-based algorithms provide compelling approaches for gene expression data analysis, effectively addressing key limitations of traditional methods (Table 1). By leveraging the within-sample rank of genes and the relative expression between gene pairs, respectively, these algorithms demonstrate notable advantages. ROGER is independent of normalization assumptions and preprocessing techniques, which mitigates the impact of technical biases. Meanwhile, PAGE excels in disease diagnosis and infection typing, demonstrating its ability to extract robust, biologically meaningful signals. Notably, PAGE reduces technical bias, enhances accuracy, facilitates cross-platform analysis, and supports functional interpretation, making it a versatile tool with broad application prospects. The unique strengths of these innovative algorithms position them as valuable additions to the gene expression analysis toolbox, with the potential to uncover new insights from complex transcriptomic datasets. As the field evolves, approaches like ROGER and PAGE, which prioritize the underlying biology over technical artifacts, are poised to play increasingly crucial roles in advancing gene expression research and clinical applications.

In conclusion, both ROGER and PAGE have demonstrated their potential as robust tools for the analysis of gene expression data, offering significant advantages over traditional normalization techniques. These methods have been successfully applied in various real-world studies, such as the development of the SepSigLnc signature for sepsis diagnosis and the GPGPS for glioma prognosis, showcasing their ability to uncover biologically meaningful insights from complex transcriptomic datasets.

Looking forward, future research could focus on further validating the applicability of these methods across a broader range of biological contexts and datasets. Additionally, there is potential for the development of more sophisticated algorithms that could integrate additional types of omics data, such as epigenetic or proteomic information, to enhance the predictive power of these methods.

**Key Points**

- CoDA restricts the quantification of NGS data, due to the library size of a sequenced sample is constrained to a constant without any biological meaning.
- ROGER is independent of the traditional normalization assumptions, which can mitigate the effect of CoDA.
- PAGE is a sophisticated strategy for data integration in either sample space or feature space of NGS data, which is also an alternative solution of CoDA.
